# Editorial: Rising stars in nutrition and food science technology: application of emerging technologies in the food industry

**DOI:** 10.3389/fnut.2023.1225703

**Published:** 2023-06-19

**Authors:** Zhi-Hong Zhang, Zhi-Wei Liu, Debao Niu

**Affiliations:** ^1^School of Food and Biological Engineering, Jiangsu University, Zhenjiang, China; ^2^College of Food Science and Technology, Hunan Agricultural University, Changsha, China; ^3^College of Light Industry and Food Engineering, Guangxi University, Nanning, China

**Keywords:** emerging technology, biological activity, food preservative, food industry, non-thermal processing

Food is essential, relating to human living and health. At present, the shortage of natural resources and environmental pressures are prominent factors in the food industry. Moreover, the demand for high-quality and natural food without chemical preservatives has been growing in recent years. In this context, the food industry faces enormous challenges and food production technologies that ensure safety and quality have become a top priority for the food industry globally. With the development of science and technology, a series of new technologies in the fields of modern nutrition, biology, optoelectronics, electromagnetism, machinery, program control, materials, and other scientific fields are widely used in scientific research and various processing links of the food industry. This can effectively improve the utilization rate of food resources and the degree of value-added processing, lead to the sustainable development of the food industry, and increase production to meet the population's growing material and nutritional needs.

In this Research Topic, the effects of emerging technologies in the food industry on food quality, microbial safety, and nutrition are summarized. Furthermore, the articles included in this RT evaluate the potential applications of these technologies in the food industry and consider current consumer demand for future food products, allowing for a deep understanding of the theory and development of these emerging technologies and how they will affect the food industry in the future.

Chinese grain vinegar is a popular daily condiment, which is produced by a solid-state fermentation style in open and non-sterile environmental conditions. In this production style, there are many shortcomings, such as the inconsistency of quality between different batches, the low production efficiency, and the low degree of mechanization. Therefore, an update to fermentation devices has been the development direction for Chinese grain vinegar in recent years. Wang W. et al. reported a solid-state fermentation technology based on a self-designed rotary drum bioreactor to study the changes in vinegar quality indexes and microbial dynamics. Results showed that the vinegar flavor changed due to the acetic acid bacteria increasing by 60% compared to the control. Moreover, the relationship between bacterial dynamics and metabolite changes in acetic acid fermentation of vinegar production was established, which can help bioreactor optimization and expand the production scale to reach industrialization.

In recent years, natural antimicrobial agents have been rapidly developed due to an increase in health and safety awareness. Antimicrobial peptides (AMPs), a class of natural antimicrobial agents that are small molecular peptides (< 100 amino acid) with antimicrobial activity and are non-toxic to humans, can be widely applied in the food industry instead of artificial synthetic preservatives. In their research article, Wang Z. et al. selected one lactic acid bacterium (named L. *plantarum* W3-2) producing bacteriocin (plantaricin W3-2) among 2,000 plant-derived strains by agar well diffusion method. The authors observed that plantaricin W3-2 showed good thermal and pH stability and broad-spectrum antimicrobial activity. Therefore, the authors suggested that this new bacteriocin is expected to become a food preservative.

In food processing, a large amount of processing waste can be produced, which could pollute the environment. Therefore, the comprehensive utilization of agricultural and food by-products is a research hotspot. Sugarcane bagasse is one of the major by-products of sugar mills. Due to the high concentration of lignin, the application of sugarcane bagasse has been limited. In their research article, Luo et al. improved the utilization rate of bagasse by applying optimal alkaline hydrogen peroxide treatment conditions (AHP). Moreover, the physicochemical properties of bagasse insoluble dietary fiber (BIDF) produced by AHP were significantly improved, such as water holding capacity, oil holding capacity, and bile salt adsorption capacity. Therefore, the authors suggested that BIDF could be used in the food industry. In another research article, Tian et al. reported that camellia shells (CSs) were used for camellia oil decolorization. The new decolorizer of the porous activated carbon-based CS was prepared by the pyrolysis plus acid (H_3_PO_4_), named CS-based p-doped porous activated carbon (CSHAC). Results showed that the adsorption efficiency of CSHAC for carotenoids was increased by 9.5%−29.5% compared to commercial decolorizers. Moreover, by analyzing the adsorption processing of carotenoids of camelia oil by CSHAC, the study showed that the adsorption property was complex adsorption, and the chemical adsorption was mainly through the adsorption process.

It is known that the maturity degree of a plant could influence the composition of chemical compounds of plant products, such as fruits, vegetables, and seeds. In their research article, Cai et al. determined the effect of different maturity stages of Noni (*Morinda citrifolia* L.) on the quality parameters of Noni polysaccharides (NP). Results showed that stages 4 and 5 have good extraction efficiency and bioactivity properties of NP, such as DPPH and ABTS free radical scavenging capacity. Therefore, the authors suggested that stages 4 and 5 could be the ideal extraction stages for obtaining high-quality Noni polysaccharides.

Compared to the traditional thermal processing technology, the non-thermal treatment technology has some advantages, such as mild treatment conditions, low-temperature rise (< 10°C), and a short treatment period. Therefore, non-thermal technology is widely studied in food sterilization to obtain good-quality food products. Wang L.-H. et al. investigated the effects of dielectric barrier discharge-air cold plasma (DBD-ACP, 15–35 kV, 2–12 min) on the quality of foxtail millets (FM). Results showed that the color of FM can be changed, and the activity of lipoxygenase and lipase was significantly decreased induced by DBD-ACP. Moreover, in the optimal treatment condition, the quality of FM was better than the control in the storage period. Therefore, the authors suggested that DBD-ACP can be an underlying approach for the storage of foxtail millets.

Due to some limitations, such as bad flavor and taste, low stability and solubility, etc., some food raw materials with nutritious value cannot be applied on a large scale in the food industry. Therefore, pretreatment is an effective method to improve their application value and health attributes. Zhang et al. studied the effect of phytochemical properties and health benefits of Tartary buckwheat flour by extrusion treatment. Results showed that pre-gelatinization of Tartary buckwheat flour by extrusion treatment can improve the antioxidant capacity, α-glucosidase inhibitory activity, and relatively mild α-amylase inhibitory activity, which suggests that it could be used as an ideal functional food resource. In another study, Ashraf et al. observed that curcumin derived from fresh rhizomes (*Curcuma longa*) by conventional (CSE) and supercritical fluid (SFE) extractions was microencapsulated using a mixture of gelatin and maltodextrin. Results showed that microencapsulation of Curcuma has better bioavailability due to its sufficient gastrointestinal residence period and stability in the digestive tract, compared to turmeric powder. Moreover, Yu et al. conducted a study to overcome astaxanthin (AST)'s serious limitations of poor water solubility and structural stability. The carrier-free astaxanthin nanoparticles (AST-NPs) were prepared, and physicochemical properties and bioactivity capacity were determined. Results showed that AST-NPs have a high loading capacity (94.57 ± 0.7%), a small average size (74.29 ± 7.92 nm), and better antioxidant activity compared to free AST. Therefore, the authors suggested that AST-NPs can become novel functional foods.

To improve the flavor and stabilize the quality of fermented food, such as fermented tea, cheese, and fermented condiment, it is necessary to study the relationship between microbial species changes and flavor ingredients during the fermentation process. In their research article, Ma et al. studied the relationship between the changes in microbial communities and volatile components of fermented minced peppers (FMP). Results showed that 17 genera were core functional microorganisms of FMP by high-throughput sequencing. Meanwhile, 64 volatile compounds were detected by GC-MS of FMP. Moreover, *Cladosporium* and *Hansenpora* were, respectively, significantly correlated with the formation of nine and six volatiles, which could guide the industrial production of FMP with unique flavors and consistent quality.

## Author contributions

Z-HZ: writing—original draft and writing—review and editing. Z-WL: writing—review and editing. DN: investigation and writing–review and editing. All authors contributed to the article and approved the submitted version.

